# Seasonal variation of microbial community and methane metabolism in coalbed water in the Erlian Basin, China

**DOI:** 10.3389/fmicb.2023.1114201

**Published:** 2023-02-10

**Authors:** Li Fu, Shouchao Lai, Zhuo Zhou, Zhenhong Chen, Lei Cheng

**Affiliations:** ^1^Key Laboratory of Development and Application of Rural Renewable Energy, Ministry of Agriculture and Rural Affairs, Biogas Institute of Ministry of Agriculture and Rural Affairs, Chengdu, China; ^2^Research Institute of Petroleum Exploration and Development, Beijing, China

**Keywords:** methanogens, methanotrophs, coalbed water, Erlian Basin, metagenomic

## Abstract

Coalbed water is a semi-open system connecting underground coalbeds with the external environment. Microorganisms in coalbed water play an important role in coal biogasification and the carbon cycle. The community assemblages of microorganisms in such a dynamic system are not well understood. Here, we used high-throughput sequencing and metagenomic analysis to investigate microbial community structure and identify the potential functional microorganisms involved in methane metabolism in coalbed water in the Erlian Basin, a preferred low-rank coal bed methane (CBM) exploration and research area in China. The results showed that there were differences in the responses of bacteria and archaea to seasonal variation. Bacterial community structure was affected by seasonal variation but archaea was not. Methane oxidation metabolism dominated by *Methylomonas* and methanogenesis metabolism dominated by *Methanobacterium* may exist simultaneously in coalbed water.

## 1. Introduction

The world’s total natural gas reserves include both conventional and non-conventional sources. Coal bed methane (CBM) is an unconventional natural gas produced during coal formation. CBM is a hydrocarbon gas that is adsorbed on the surface of coal matrix particles and dissociated in coal pores or dissolved in the water of coal seams (Moore, 2012). Methane in coal is derived from both biogenic and thermogenic processes (Strapoc et al., 2011), and approximately 20% of the world’s natural gas is biogenic (Wang et al., 2016). The origin of methane depends upon the maturity of coal. Generally, deep and highly matured coal is associated with thermogenic methane, whereas shallow and less mature coal is associated with biogenic methane. Coal reservoirs of moderate depth and maturity have a mixture of biogenic and thermogenic methane.

The biogasification of coal is a mixed fermentation process involving many different microorganisms (Faiz and Hendry, 2006; Park and Liang, 2016). Bacteria related to Firmicutes, Actinobacteria, and Bacteroidetes and all subgroups of Proteobacteria seem to be widespread in CBM (Beckmann et al., 2011a; Colosimo et al., 2016). These taxonomic groups are known for their versatile metabolic activity and hydrocarbon degrading capabilities. In the presence of microorganisms, coal (complex polymers) is degraded into a series of intermediates such as polycyclic aromatic hydrocarbons (PAHs), heterocyclic compounds, alkyl phenols, aromatic amines, alkyl aromatics, long-chain fatty acids, and aliphatic hydrocarbons (Park and Liang, 2016; Wang et al., 2018). These are intermediates to the formation of acetate and H_2_, which undergo methanogenesis (Conrad, 2020). Methanogens commonly detected in coal seams environments can be divided into three types according to their methanogenic pathways. Hydrogenotrophic methanogens like *Methanobacterium* consume H_2_ to reduce CO_2_ to generate CH_4_ (Rosewarne et al., 2013). Acetotrophic methanogens like *Methanosarcina* and *Methanosaeta* convert acetate into CH_4_ and CO_2_ (Krüger et al., 2008; Beckmann et al., 2011b). Methylotrophic methanogens like *Methanolobus* use methanol, methylamine, and other methyl substances to produce CH_4_ (Doerfert et al., 2009; Guo et al., 2012b). Unexpectedly, Mayumi et al. characterized a strain of *Methermicoccus shengliensis* that can produce methane from the dozens of methoxylated aromatic compounds found in a variety of coal types (Mayumi et al., 2016). This portends methane production from coal by a single methoxydotrophic methanogen. Methane in these environments can be utilized by methanotrophs as electron donors and energy sources (Smith and Wrighton, 2019). Methanotrophic microorganisms play roles in mitigating methane emissions. However, the function of these microorganisms is often neglected in studies of CBM.

The Erlian Basin investigated in this study has CBM geological characteristics similar to the Powder River basin in the United States (Ayers, 2002; Sun et al., 2017), and is the preferred low-rank CBM exploration and research area in China (Chen and Pan, 2019). The resource quantity of low-rank CBM in the Erlian Basin is approximately 2.48 × 10^12^ m^3^ (Sun et al., 2018). In order to effectively develop low-rank CBM in China, a simulation experiment was conducted to test the microbial community and clarify the methanogenic pathway and biological gas recovery potential in the coal seams of the Jiergalangtu depression, Erlian Basin. The results showed that both aceticlastic and hydrotrophic methanogenesis were detected in the coal and coalbed water (Sun et al., 2018). Previous studies have explored the methane production potential (Lai et al., 2020) and influencing factors (Chen et al., 2018) of coalbed organisms in the Erlian Basin. However, the above studies were based on simulation experiments, and cannot reflect the remodeling of microbial community structure that occurs through real environmental changes.

Coalbed water is a semi-open system connecting the underground coalbed with the external environment, and the microorganisms in coalbed water respond directly to various environmental changes. Their response mechanism is of great importance to both microbial mechanism research and production applications. In recent years, the application of high-throughput sequencing (Guo et al., 2012a; Wei et al., 2014; Beckmann et al., 2019) and metagenomics (Robbins et al., 2016) have gradually revealed the biogenic CBM microbial community. Metagenomics allows to assess the presence and genomic potential of an organism, from which hypotheses about its physiology can be derived. Therefore, in this study, coalbed water samples were collected from the Erlian Basin during different seasons. The seasonal variation in the microbial community and the functional microorganisms involved in methane metabolism were investigated using high-throughput sequencing and metagenomic analysis.

## 2. Materials and methods

### 2.1. Coalbed water sample collection

Twenty-three original samples of coalbed water were collected from the Erlian Basin (Supplementary Figure S1) in May, August, and October 2018, corresponding to the local spring, summer, and autumn. Meteorological conditions such as precipitation and temperature vary greatly between seasons in this region. In May, August, and October 2018, the total monthly precipitation was 6.6, 30.3, and 0.7 mm, and the mean temperature was 18.2, 23.1, and 4.4°C, respectively (Supplementary Table S2). The formation water samples were gathered from a depth of 400 meters. Water samples dedicated for chemical composition analysis were kept on ice. The basic physical and chemical properties of coalbed water were determined by the unconventional experimental center of China National Offshore Oil Corporation Energy Development Co., LTD. The pH of the samples ranged from 7.53 to 8.79, which was slightly alkaline. Nitrite ions were detected in only two samples (JM10O and JM13O). Nitrate and sulfate concentrations were also detected at low levels. Sodium concentration was detected at a high level based on the Chinese classification of mine water (Supplementary Table S1). Water samples dedicated for microbial analysis were stored and transported in ambient temperature. Water samples were transported by road, and the average transportation time was 3 days.

### 2.2. DNA extraction

For original coalbed water samples, 500 ml water sample was required per sample for high speed centrifugation (5 min at 14,000 *g* at 4°C). For the sample of the culture experiment, 2 ml of culture at the end of the experiment was required for high speed centrifugation (5 min at 14,000 *g* at 4°C). After the supernatant was discarded, the precipitate was resuspended with 20 ml phosphate buffer, and the suspension was transferred to a new, enzyme-free spiral tube for further centrifugation. The precipitate obtained after final centrifugation was stored at −80°C until DNA extraction. DNA was extracted using a Water DNA Isolation Kit (Foregene).

### 2.3. High-throughput sequencing of the 16S rRNA genes of bacteria and archaea

High-throughput sequencing was performed on the extracted DNA samples by Beijing Nuohe Zhiyuan Co. Ltd. DNA concentration and purity were determined by ultramicro-spectrophotometer, and the quality of all DNA samples met the sequencing requirements. The V3-V4 region of the bacterial 16S rRNA genes was amplified using the bacteria-specific primers 341F_CCTAYGGGRBGCASCAG/806R_GGACTACNNGGGTATCTAAT. The V4-V5 region of archaea 16S rRNA genes was amplified using the archaea-specific primers Arch519F_CAGCCGCCGCGGTAA/Arch915R_GTGCTCCCCCGCCAATTCCT. Illumina HiSeq 2,500 was used for pair-end sequencing after the amplification products were recovered by gel cutting.

### 2.4. High-throughput data analysis

QIIME software was used to conduct quality control, chimeric removal, re-sampling, OTU (Operational Taxonomic Unit) clustering, selection of representative sequences, species annotation, and singleton removal for sequencing data from the sequencing unloading machine. Because of limited primer specificity, a small number of archaea sequences were found in the amplified fragments of bacterial primers, and bacterial sequences were also amplified by archaea primers. After the mismatched OTUs were deleted by Excel, the structure of the microbial community was analyzed. For original coalbed water samples, a total of 673,216 high-quality sequences were obtained using high-throughput sequencing, among which 502,724 were bacterial sequences and 170,492 were archaeal sequences (Supplementary Table S3). A total of 1,009 archaeal OTUs were obtained based on a 97% sequence similarity and the average number of OTUs of the samples was 44. The number of OTUs in samples JM3A (104) and JM4-1A (102) was higher than that of other samples. A total of 20,082 bacterial OTUs were obtained, and the average number of OTUs per sample was 873. The Shannon index indicated high bacterial diversity (3.57 ± 0.51; mean ± SD). Coverage for bacteria and archaea ranged from 0.96 to 0.98, indicating that sequencing depth was sufficient.

### 2.5. Metagenomics sequencing and analysis

The genomic DNA was randomly sheared into short fragments. The obtained fragments were end repaired, A-tailed and further ligated with Illumina adapter. The fragments with adapters were PCR amplified, size selected, and purified. The library was checked with Qubit and real-time PCR for quantification and bioanalyzer for size distribution detection. Quantified libraries will be pooled and sequenced on Illumina platforms, according to effective library concentration and data amount required. The raw reads were dereplicated and quality-trimmed using Trimmomatic (Bolger et al., 2014). High-quality metagenomic sequences were *de novo* assembled using SPAdes (v.3.12.0) with the default k-mer size (Nurk et al., 2017). The assembled scaffolds were binned using MetaBAT (v.2.12.1) based on tetranucleotide frequency, GC content, and coverage (Kang et al., 2019). Partial and near-complete genomes were recovered after binning. The completeness, contamination, and strain heterogeneity of MAGs were evaluated using CheckM (v.1.0.11) based on lineage-specific marker sets (Parks et al., 2015). The taxonomic assignment of the different bins was conducted with the GTDB-Tk package (v.1.3.0; Chaumeil et al., 2019), and bins were translated by Prodigal using the “-p meta” parameters (Hyatt et al., 2010). For each predicted coding sequence (CDS), protein function was annotated using the KEGG server (BlastKOALA) and eggNOG-mapper (Kanehisa et al., 2016).

### 2.6. Methanogenic culture experiment

Water sample JM13M was selected for methanogenic simulation culture. After shaking well, the 180 ml water sample was added to the 300 ml sterilized and dried serum bottle. The pH was adjusted to neutral. Na_2_S·9H_2_O (1.0 mM), resazurin (0.0005 g L^−1^), trace elements (2.0 ml/L), and vitamins (V284/VB1/VB12; 4.0 ml/L) were supplemented. Vitamin and trace element solutions were prepared as described previously (Lu and Lu, 2012). The headspace gas was flushed with N_2_ for 30 min to remove oxygen. Fourteen methanogenic precursors were selected for methanogenic simulation culture:H_2_/CO_2_ (50 ml/25 ml), sodium formate (20 mM), dimethyl sulfide (20 mM), dimethylamine (20 mM), methanol (20 mM), sodium acetate (20 mM), sodium propionate (20 mM), sodium butyrate (20 mM), ethanol (20 mM), 1-propanol (20 mM), 2-propanol (20 mM), 1-butanol (20 mM), 2-butanol (20 mM), and Choline hydroxide (20 mM). No precursor was added to the control. Three parallel were set for each treatment and incubated at 35°C in the dark.

### 2.7. CH_4_ detection

The CH_4_ content in the headspace of all culture bottles was measured using gas chromatograph 7820A (Agilent Technologies, United States) with Porapak Q capillary columns (3 m) at 65°C. High-purity hydrogen (99.999%) was used as the mobile phase. The column flow rate was 27 ml/min. TCD (thermal detector) at 130°C, the inlet temperature was 105°C, the reference flow was 30 ml/min, and the tail blow flow was 2 ml/min. The gas detection time was 1.5 min, and the injection volume was 0.2 ml. The relative proportions of N_2_, CH_4_, and CO_2_ in the sample gas were calculated using the corrected area normalization method for the peak diagram. All samples were tested at 5-day intervals.

### 2.8. Data availability

The datasets generated for this study have been deposited under NCBI. For original coalbed water samples, 16S rRNA genes of archaea: PRJNA681683. 16S rRNA genes of bacteria: PRJNA681635. For the sample of the culture experiment, 16S rRNA genes of archaea and bacteria: PRJNA923302. Metagenomic raw data: PRJNA682244 (Supplementary Table S9).

## 3. Results and discussion

### 3.1. Response of microbial community structure to seasonal variation

**Figure 1:**
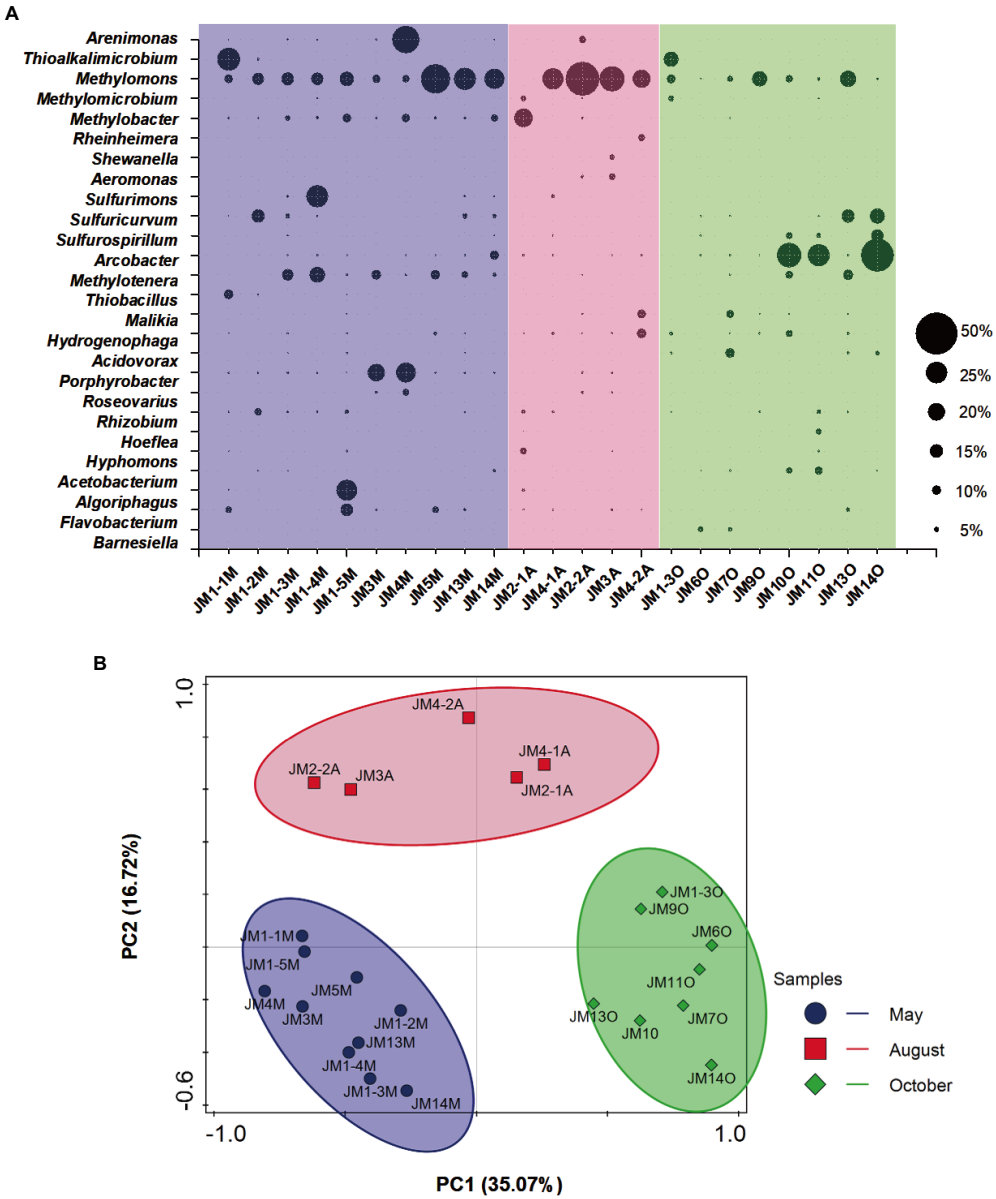
**(A)** Responses of bacterial community structure to seasonal variation in coalbed water based on 16S rRNA gene. The suffix letter of each sample name represents the month in which the sample was taken (M: May; A: August; O: October). The size of the bubble represents the relative abundance of the genus in each sample. **(B)** Principal components analysis (PCA) of bacterial 16S rRNA gene retrieved from coalbed water. Different symbols and colors represent different sampling months (purple circle: May; red square: August; green diamond: October).

A total of 24 bacterial phyla, including the candidate divisions, were detected from coalbed water samples (Supplementary Figure S2). Proteobacteria was the most abundant phylum, and the average relative abundance of Proteobacteria in the 23 samples was 77.3% (range 25.6–93.0%). Other bacteria were mainly affiliated with the phyla Bacteroidetes (10.1%) and Firmicutes (6.7%; Supplementary Figure S2). Bacteria with a relative abundance of more than 5% (genus level) in each sample were selected, as shown in Figure 1A. The dominant Gammaproteobacteria genera were *Methylomonas* (15.1%), *Methylobacter* (2.8%), *Thioalkalimicrobium* (2.1%), *Arenimonas* (2.0%) *Methylomicrobium* (0.6%), *Aeromonas* (0.4%), *Rheinheimera* (0.3%), and *Shewanella* (0.3%). Epsilonproteobacteria included *Arcobacter* (5.2%), *Sulfuricurvum* (2.9%), *Sulfurimonas* (1.5%), and *Sulfurospirillum* (1.3%). Betaproteobacteria included *Methylotenera* (3.8%), *Hydrogenophaga* (1.8%), *Acidovorax* (1.1%), *Malikia* (1.1%), and *Thiobacillus* (0.6%). Alphaproteobacteria included *Porphyrobacter* (2.5%), *Rhizobium* (1.3%), *Roseovarius* (0.7%), *Hyphomonas* (0.6%), and *Hoeflea* (0.4%). Bacteroidetes included *Flavobacterium* (1.8%), *Algoriphagus* (1.3%), and *Barnesiella* (0.5%). In Firmicutes, only *Acetobacterium* (1.1%) showed slightly higher relative abundance.

OTUs with a relative abundance of >5% included primarily methanotrophs, methylotrophs, acetogenic bacteria, chemoautotrophic bacteria, and fermentative bacteria. The aerobic methanotrophs were *Methylomonas* and *Methylobacter*, and *Methylomonas* dominated the coalbed water with the highest relative abundance (up to 40%; Figure 1A). Methylotrophic bacteria included *Methylotenera*, whose substrate spectrum is narrow and could only metabolize methylamine, methanol, and other methyl compounds (Kalyuzhnaya et al., 2012; Mustakhimov et al., 2013). *Acetobacterium* can generate acetic acid using H_2_/CO_2_, short-chained alcohols, and various carbohydrates (sugars; Kremp et al., 2018; Westphal et al., 2018). Chemoautotrophic bacteria included *Thiobacillus*, *Sulfurospirillum*, *Sulfurimonas*, and *Sulfuricurvum*. *Thiobacillus* (Kellermann and Griebler, 2009), *Sulfurospirillum* (Stolz et al., 1999; Luijten et al., 2003; Hubert and Voordouw, 2007), and *Sulfurimonas* (Inagaki et al., 2003; Sievert et al., 2008) were the non-strict chemoautotrophic bacteria, which utilize reductive sulfide, hydrogen, and organic matter (glucose, volatile fatty acid, amino acid, etc.) as electron donors, nitrate or sulfate as electron acceptors, and carbon dioxide as a carbon source. *Sulfuricurvum*, however, is a strictly chemoautotrophic bacteria, and can only use hydrogen and reductive sulfides as an electron acceptor.

PCA results showed that the bacterial community structure of coalbed water samples in the Erlian Basin was clustered according to sampling months. There was no overlap or crossover between the bacterial communities in the coalbed water during the three sampling periods (Figure 1B). This indicated that the community structure of bacteria in coalbed water in this block differed among seasons. A large number of aerobic bacteria (*Methylomonas*, *Methylobacter*, *Methylotenera*, *Sulfurospirillum*, *Acidovorax*, and *Thioalkalimicrobium*) were detected in the coalbed water, which may be related to atmospheric precipitation. Atmospheric precipitation leads to surface runoff into coal seams and brings in oxygen, nutrients, and electron receptors. However, the periodicity of rainfall leads to an unsustainable supply of oxygen, while the metabolic activity of aerobic microorganisms causes periodic changes in oxygen concentration, resulting in co-existence of aerobic and anaerobic bacteria in coalbed water (Bates et al., 2011). The meteorological data from the National Meteorological Science Data Center^1^ show that there were significant differences in rainfall and mean temperature among the sampling sites during different seasons (Supplementary Table S2). However, due to the lack of reliable measured data, we are still unable to attribute bacterial community changes.

**Figure 2:**
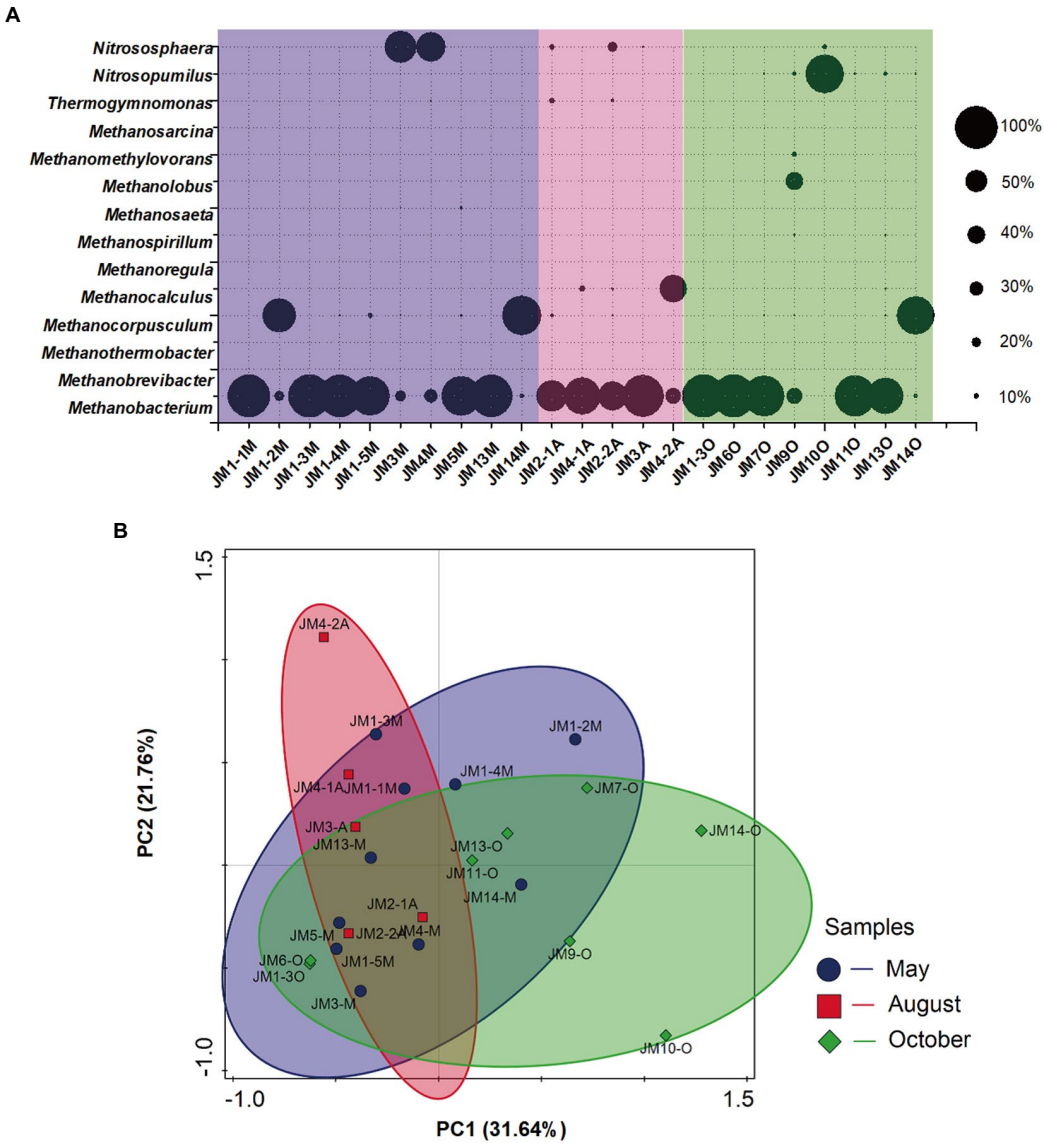
**(A)** Responses of archaeal community structure to seasonal variation in coalbed water based on 16S rRNA sequencing. The suffix letter of each sample name represents the month in which the sample was taken (M: May; A: August; O: October). The size of the bubble represents the relative abundance of the genus in each sample. **(B)** Principal component analysis (PCA) of archaeal 16S rRNA gene retrieved from coalbed water. Different symbols and colors represent different sampling months (purple circle: May; red square: August; green diamond: October).

At the phylum level, archaea were dominated by *Euryarchaeota* (relative abundance 74.6–92.3%). Only some of the archaea in samples JM2 and JM9 were distributed in *Nanoarchaeota* (relative abundance 8.1–8.6%) and *Thaumarchaeota* (relative abundance 5.2–6.9%). At the genus level, the dominant archaea were hydrogenotrophic methanogens *Methanobacterium*, and the average relative abundance of the 23 samples was 61.6% (range 6.6–98.2%; Figure 2A). In addition, the dominant archaea in sample JM4A were hydrogenotrophic methanogens *Methanocalculus* (relative abundance 62.9%), and the dominant archaea in JM11O, JM1_2M, and JM14M were hydrogenotrophic methanogens *Methanocorpusculum*, with relative abundances of 87.7, 77.0, and 87.9%, respectively. The methylotrophic *Methanolobus* (Doerfert et al., 2009; relative abundance 36.9%) in JM9O was also relatively high.

PCA showed that the archaea communities of the 23 coalbed water samples were partially overlapping and were dispersed in different areas in the PCA diagram, with no obvious clustering (Figure 2B). This indicated that the community structure of archaea in coalbed water in this block was relatively stable. The archaea community structure is affected by many environmental factors. All methanogens are strict anaerobes, not documented to use O_2_ as an electron acceptor during energy-conserving metabolism (Horne and Lessner, 2013). Nutrient availability is one of the key factors determining the abundance of methanogens (Zhou et al., 2015). Salinity (Waldron et al., 2007; Zhang et al., 2020) and temperature (Lu et al., 2015) are also primary environmental factors regulating methanogenic community assemblage. The different responses of archaea and bacteria to seasonal variation in coalbed water reflect their distinct ecological niches.

### 3.2. Methane production potential of microorganisms In coalbed water

**Figure 3:**
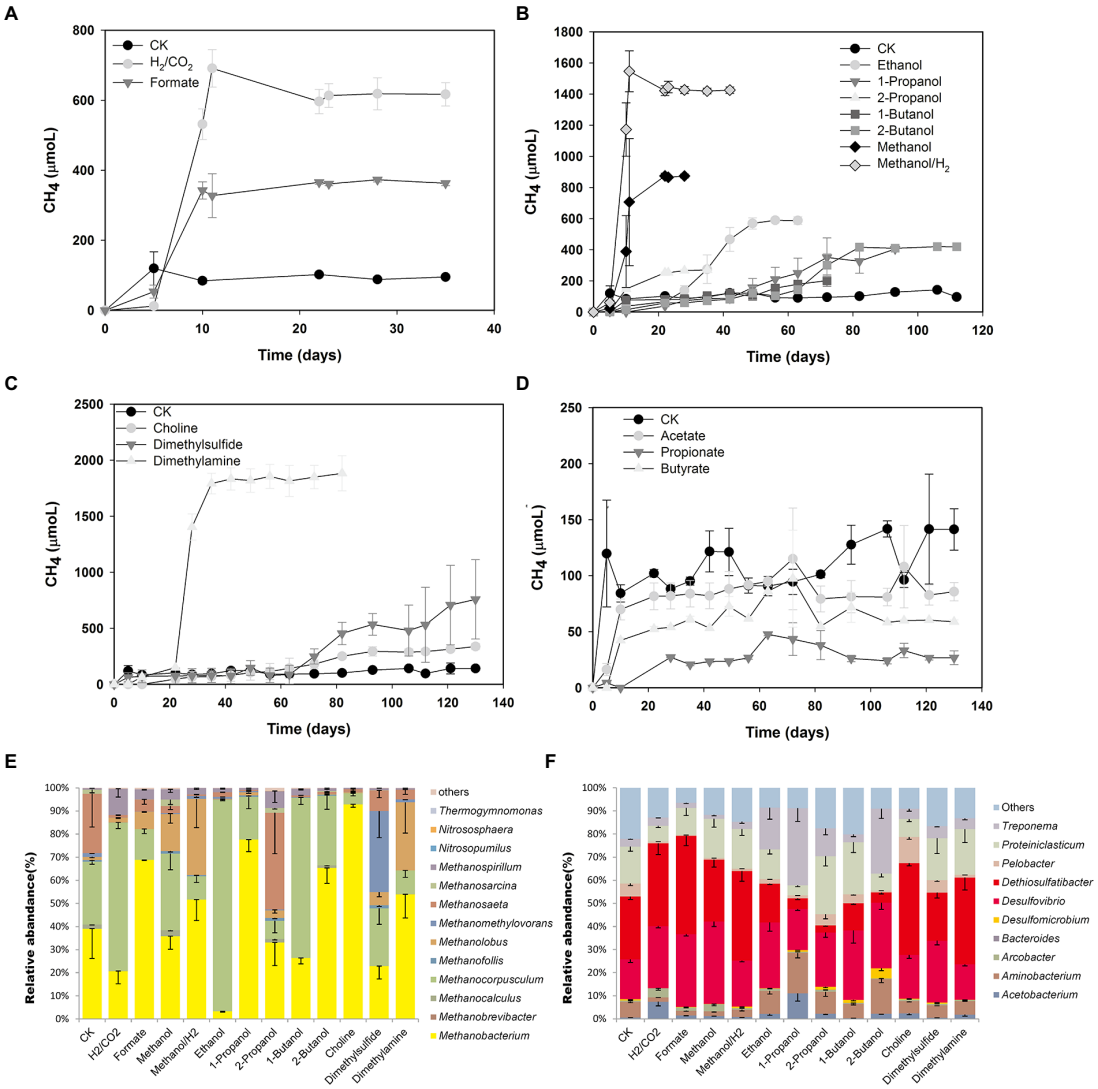
Methane production potential and microbial community structure of different methane precursors. Methane production from H_2_/CO_2_ and formate **(A)**, short-chain fatty alcohols **(B)**, methyl compounds **(C)**, and short-chain fatty acids **(D)**. The relative abundances of archaeal **(E)** and bacterial **(F)** 16S rRNA genes.

In order to verify the methane production potential of microorganisms in coalbed water and assess its methanogenic pathway, coalbed water sample JM13M was selected to study methanogenic potential using different precursor compounds. H_2_/CO_2_ and formate (Figure 3A), methyl compounds (methanol, dimethylamine, dimethyl sulfide, choline; Figure 3C), and short-chain fatty alcohols (propanol, isopropyl alcohol, butanol, and isobutanol; Figure 3B) may be used by microorganisms in JM13M to produce CH_4_, while short-chain fatty acids cannot be used (Figure 3D). Both the lag phase and the specific growth rate of methane production were different under different substrate treatment (Supplementary Table S4). The dominant archaea were the hydrogenotrophic methanogens *Methanobacterium* (relative abundance 2.9–91.7%) and *Methanocorpusculum* (relative abundance 4.9–91.6%), and the methylotrophic *Methanolobus* (relative abundance 1.2–32.9%; Figure 3E). The methanogenic activity of coalbed water was confirmed by the simulation culture (Figure 3). The bacterial community mainly consisted of *Desulfomicrobium* and *Desulfovibrio* (Figure 3F), which might be related to the addition of sodium sulfide.

**Figure 4:**
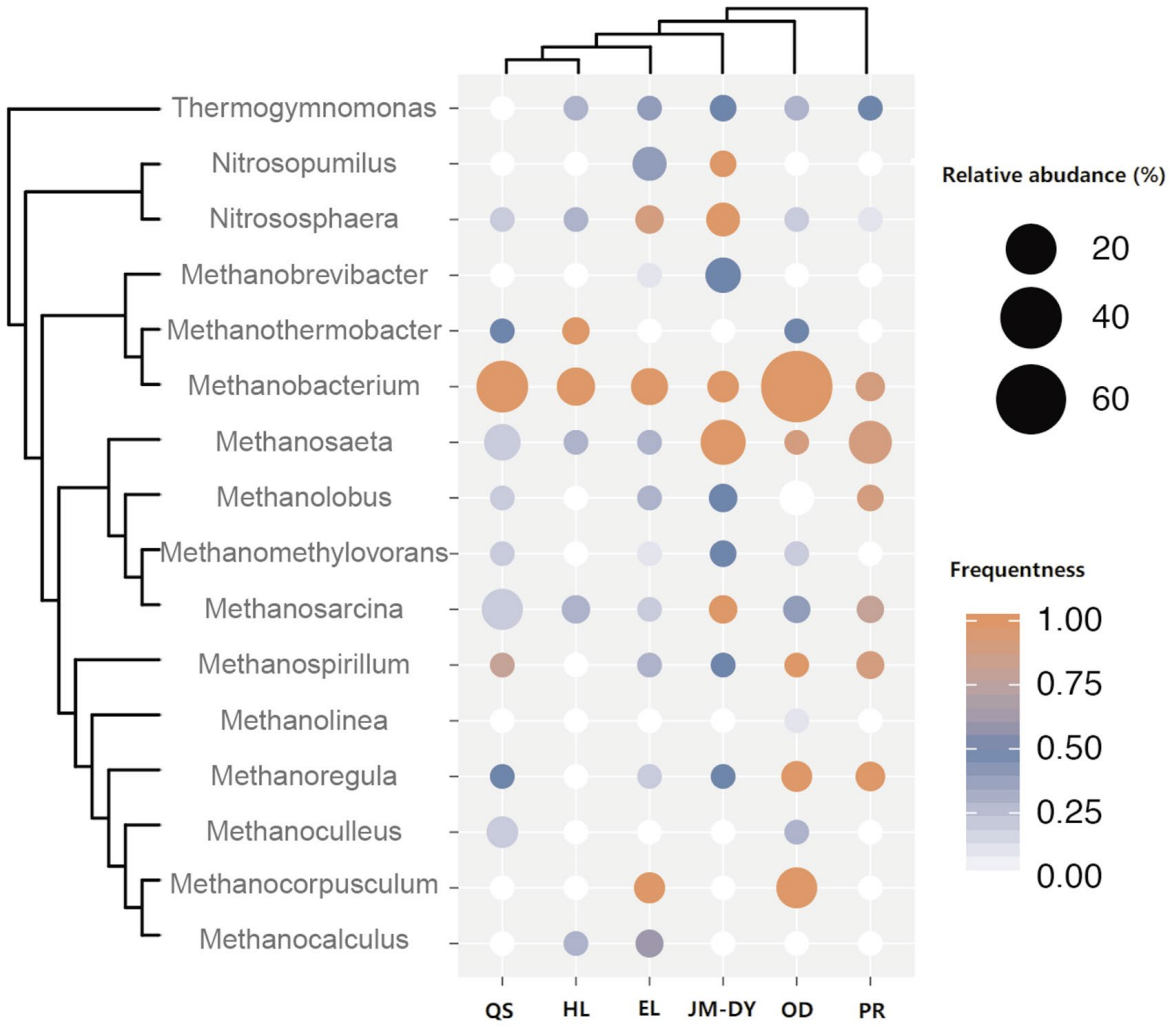
Comparative analysis of archaea community structure in coal seams in different basins (QS: Qinshui Basin; HL: Hailar Basin; EL: Erlian Basin; JM-DY: Jingmen-Dangyang Basin; OD: Ordos Basin; PB: Powder river Basin). The size of the bubble represents the relative abundance of the genus in each sample. The change in bubble color represents the frequency of the genus detected.

We also collected published and available coalbed archaea data from different basins, including Qinshui Basin (Guo et al., 2017; Yang et al., 2018), Hailar Basin, Jingmen-Dangyang Basin (Wei et al., 2014), Ordos Basin (Guo et al., 2012a), and Powder river Basin (Huang et al., 2017; Davis et al., 2018), and combined them with the Erlian Basin data for analysis (Figure 4). We found that most of these basins were dominated by hydrogenotrophic methanogens *Methanobacterium* (except the Powder River basin and JM-DY Basin, which were dominated by acetotrophic methanogens). *Methanobacterium* is widely distributed in anaerobic habitats such as marine and freshwater sediments, soils, animal gastrointestinal tracts, anaerobic sewage digesters, and geothermal habitats (Liu, 2010). *Methanobacterium* are typically autotrophic and use H_2_ and CO_2_, while some also use formate as a substrate for methanogenesis (Liu, 2010). Formate dehydrogenases that supply reductant from formate oxidation and that yield intracellular CO_2_ to allow for methanogenesis to proceed under otherwise dissolved inorganic carbon (DIC) limited conditions (Fones et al., 2021). Recent studies have shown that *Methanobacterium* are also able to engage in direct interspecific electron transfer (Zheng et al., 2020), which facilitates the formation of a more efficient connection between *Methanobacterium* and syntrophic bacteria. These properties allow *Methanobacterium* to thrive in a variety of methane-producing environments, including coalbed water. *Methanocorpusculum* was also dominant in some coalbed water samples, and was the most prominent genus in a coal bed of the Illinois Basin (Strapoc et al., 2008) and in shale in northern Michigan (Waldron et al., 2007). *Methanocorpusculum* belong to the order *Methanomicrobiales* within the archaeal phylum *Euryarchaeota*. Similar to *Methanobacterium*, *Methanocorpusculum* use either H_2_/CO_2_ or formate as substrate for methanogenesis. Compared to other strains of *Methanomicrobiales*, *Methanocorpusculum labreanum* was the only strain showing evidence of genome downsizing (Browne et al., 2017), which leads to niche specialization (Lee and Marx, 2012). However, *M. labreanum*’s putative specialization remains unclear.

### 3.3. Aerobic methane-oxidizing bacteria in coalbed water presented by metagenomics

The previous high-throughput sequencing results revealed high relative abundance of aerobic methane-oxidizing bacteria in coalbed water. This implied that the methane oxidation reaction was very active. However, the mechanisms by which aerobic methane-oxidizing bacteria act in coalbed water warranted further study. For this, three coalbed water samples (JM1-2M, JM5M, and JM13M) were selected for metagenomic sequencing (Supplementary Table S5). After sequencing and quality control of the sequences, taxonomic classification was performed using two different tools, Kraken2 and MetaPhlAn2. Overall, bacteria (relative abundance >1%) accounted for 60.0–76.9% of the genera identified by Kraken2 and 96.7–97.4% of the genera identified by MetaPhlAn2 (Supplementary Figure S3). However, the relative abundance of archaea in metagenomic data was too low (<0.02%), as shown in the figure.

Kraken2 and MetaPhlAn2 produced similar mock community composition profiles. According to Kraken2, *Methylomonas* (19.0–51.7%) occurred in a higher proportion in bacteria (Supplementary Figure S3B), and the same result was found using MetaPhlAn2 (51.8–87.1%; Supplementary Figure S3A). Such a high abundance of *Methylomonas* was also consistent with our high-throughput sequencing results. In addition, *Methylotenera*, *Pseudomonas*, and *Thiomonas* occupied a certain proportion in some samples. Among them, *Pseudomonas* was particularly noteworthy due to its unique value to research and application to PAH and coal biodegradation (Foght and Westlake, 1988; Tang et al., 2011; Poblete-Castro et al., 2012).

Metagenomic data were used to assemble methane-oxidizing bacteria genomes. Seven high-quality genome bins (MAGs) were obtained (Supplementary Table S6), and after dereplication with dRep with an ANI cutoff of 97%, five cluster MAGs were obtained for subsequent analysis (Supplementary Table S7). Phylogenetic trees constructed with the genomes of methane-oxidizing bacteria (Supplementary Table S8) showed three clusters affiliated with *Methylomonas* (Supplementary Figure S4).

**Figure 5:**
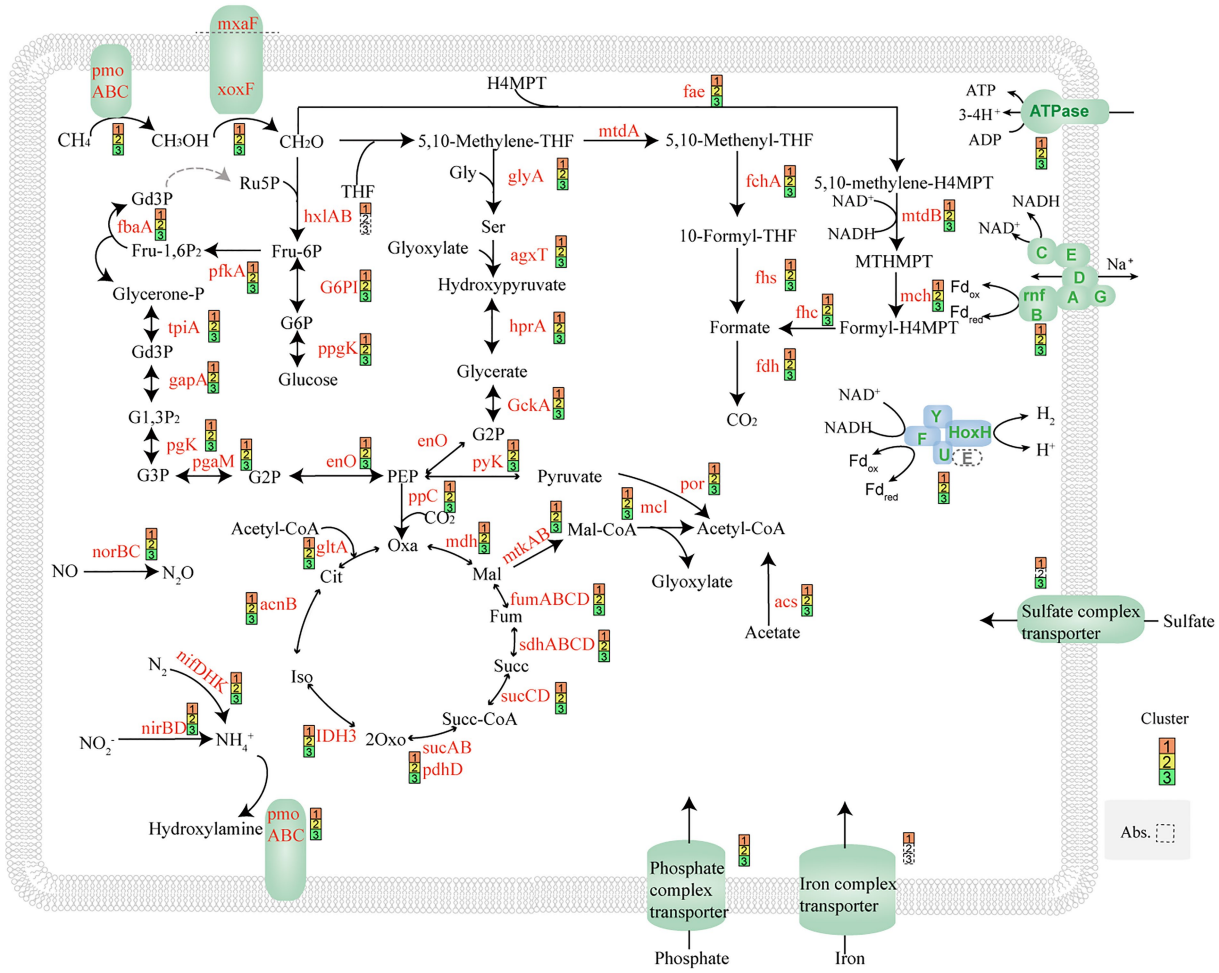
Proposed pathway by MAGs of *Methylomonas* based on metagenomics analyses. All MAGs use pmo complex to conduct the oxidation of methane to methanol. XoxF and mxaF dehydrogenase, which can catalyze methanol to formaldehyde, were found. In this study, MAGs have complete RuMP cycles, serine cycles, EMP, and TCA cycle pathways. NifDHK nitrogenase, NiRk dissimilatory nitrite reductase, NorB nitric oxide reductase, and HoxHY hydrogenase were also found in MAGs.

These three cluster MAGs contained complete metabolic pathways for aerobic methane oxidation. In the first step of methane metabolism, methane is captured and converted to methanol by granular methane monooxygenase (pMMO), an enzyme complex that uses oxygen to oxidize the C-H bonds in methane. In the second step of methane oxidation, methanol is catalyzed by methanol dehydrogenase (MDH) to form formaldehyde. There are two known MDH enzymes, the canonical and long-studied MxaF type and a novel type, XoxF (Chistoserdova et al., 2009). These three cluster MAGs encode both types of methanol dehydrogenases. In the next step, formaldehyde is a key metabolite. Formaldehyde can be converted to formate *via* the tetrahydromethanopterin (H_4_MPT) pathway or the methylene-tetrahydrofolate (methylene-H_4_F) pathway. Another part of formaldehyde can be metabolized through the RuMP cycle and the serine cycle. All genes coding for these pathways were detected in our MAGs (Figure 5). Succinate dehydrogenase (complex II, sdhABCD) was found in the MAGs, together with genes encoding cytochrome *c* oxidase. The proton motive force generated by the respiratory chain can be used by ATPase (complex V). Nitrogenase (NifDHK), dissimilatory nitrite reductase (NiRk), and nitric oxide reductase (NorB) were also found in these MAGs. This means that these genomes may have the ability to fix nitrogen and convert nitrite into ammonium through denitrification (Figure 5). Denitrification may support methane oxidation in environments with low or below-detectable oxygen concentrations (Padilla et al., 2017; Smith et al., 2018; Smith and Wrighton, 2019).

## 4. Conclusion

Microbial gas recovery is one of the effective means to improve the efficiency of low-rank CBM recovery. Low-rank CBM accumulation and coal reservoir characteristics are controlled by not only geological factors such as structure, coal rank, and hydrology, but also microbial factors. In this study, seasonal variation in microbial communities and the functional microorganisms involved in methane metabolism in Erlian Basin were investigated. There were differences in the responses of bacteria and archaea to seasonal variation. The bacterial community structure in coalbed water was affected by seasonal variation. The archaea community structure did not change with seasonal variation. *Methylomonas* may play an important role in methane oxidation metabolism in coalbed water. *Methanobacterium*, the dominant hydrogenotrophic methanogens, may play a dominant role in methanogenic metabolism. Further research should focus on the driving factors that cause seasonal variation of bacterial community structure. This will provide some theoretical basis for the regulation of methane metabolism of low-rank CBM in this basin.

## Data availability statement

The original contributions presented in the study are publicly available. These data can be found in GenBank, 16S rRNA genes of archaea: PRJNA681683, 16S rRNA genes of bacteria: PRJNA681635, and Metagenomic raw data: PRJNA682244 and PRJNA923302 (Supplementary Table S8).

## Author contributions

LC and ZC conceived the research. SL performed the sample collection, *in situ* simulated incubation, and molecular analysis. ZZ performed the Metagenomics analysis. LF wrote the manuscript. LF and LC edited the manuscript. All authors reviewed and approved the manuscript. All authors contributed to the article and approved the submitted version.

## Funding

This work was supported by the National Key R&D Program of China (2016ZX05041001-003), Agricultural Science and Technology Innovation Project of Chinese Academy of Agricultural Sciences (CAAS-ASTIP-2016-BIOMA), and the Central Public-interest Scientific Institution Basal Research Fund (No. 1610012020006-03104).

## Conflict of interest

The authors declare that the research was conducted in the absence of any commercial or financial relationships that could be construed as a potential conflict of interest.

## Publisher’s note

All claims expressed in this article are solely those of the authors and do not necessarily represent those of their affiliated organizations, or those of the publisher, the editors and the reviewers. Any product that may be evaluated in this article, or claim that may be made by its manufacturer, is not guaranteed or endorsed by the publisher.

## Footnotes

^1^ http://data.cma.cn/
