# Supplementary material for: Seasonal variation of microbial community and methane metabolism in coalbed water in the Erlian Basin, China
Source: Front Microbiol. 2023 Feb 10;14:1114201. doi: 10.3389/fmicb.2023.1114201 (PMC9953142; doi:10.3389/fmicb.2023.1114201)
Supplement: Supplementary file 2 [file Data_Sheet_1.docx]

***Supplementary Material***

**Seasonal variation of microbial community and methane metabolism in** **coalbed water in the Erlian basin, China**

**Li Fu^1^, Shouchao Lai^1^, Zhuo Zhou^1^, Zhenhong Chen^2*^** **and Lei Cheng^1*^**

^1^Key Laboratory of Development and Application of Rural Renewable Energy, Ministry of Agriculture and Rural Affairs, Biogas Institute of Ministry of Agriculture and Rural Affairs, Chengdu, China

^2^Research Institute of Petroleum Exploration and Development, Beijing, China

***Corresponding author:**

Zhenhong Chen

Research Institute of Petroleum Exploration and Development, Beijing 100083, PR China

Phone/Fax: +86-10-83593123

E-mail: chenzhenhong@petrochina.com.cn

Lei Cheng

Key Laboratory of Development and Application of Rural Renewable Energy, Ministry of Agriculture and Rural Affairs, Biogas Institute of Ministry of Agriculture and Rural Affairs, Chengdu 610041, PR China

Phone/Fax: +86-28-85215106

E-mail: chenglei@caas.cn

**Running title**: Succession of microbial community structure in coalbed water

**Keywords**: Methanogens, methanotrophs, coalbed water, Erlian basin, metagenomic


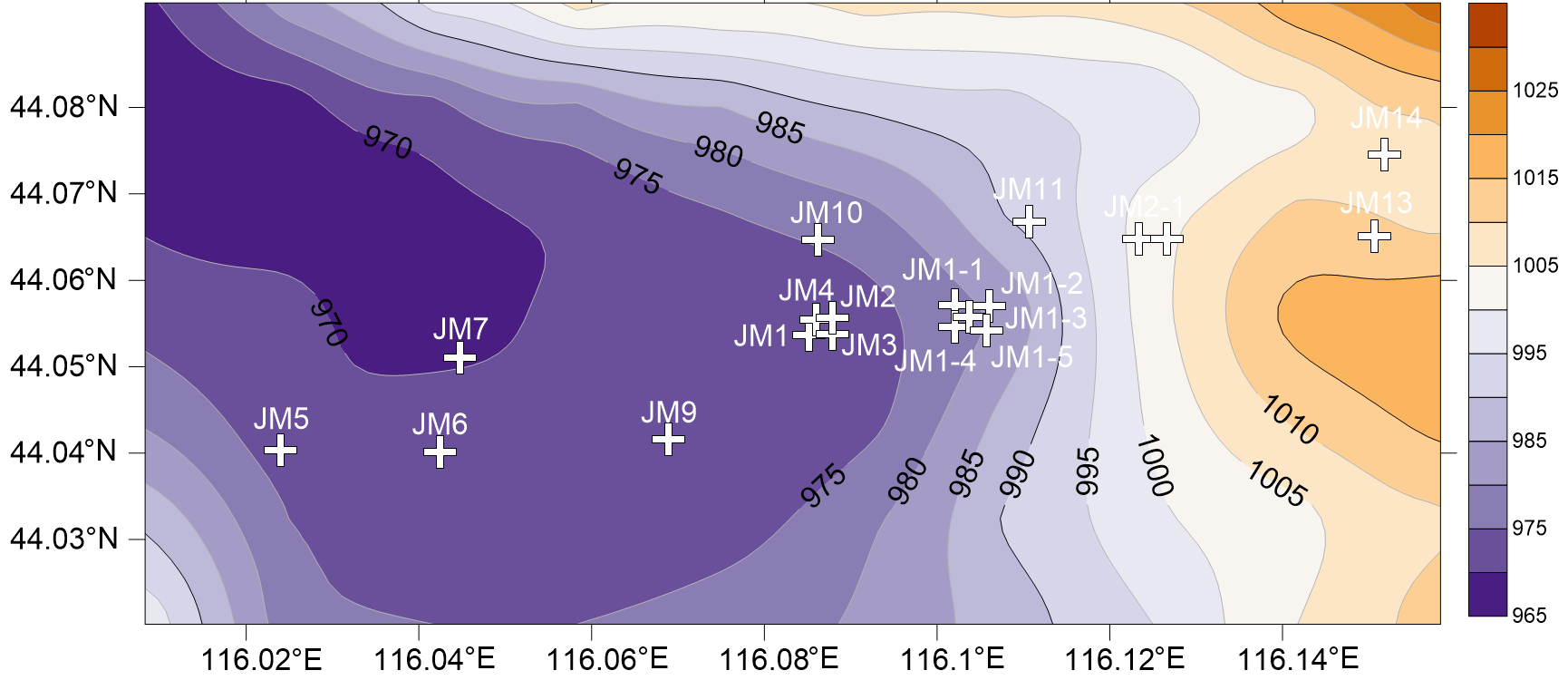
Figure S1. Map of the Erlian Basin, showing location of the sampled well.


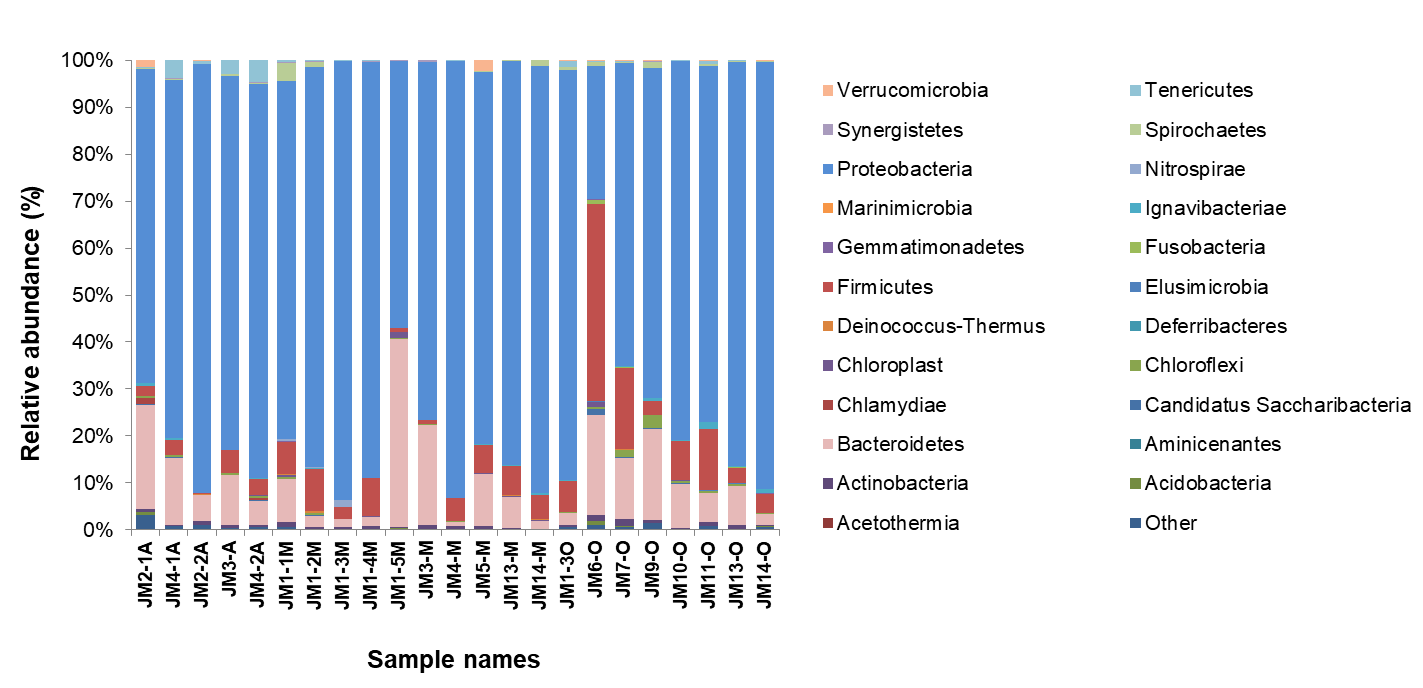
 Figure S2. Bacterial community structure at phylum level.


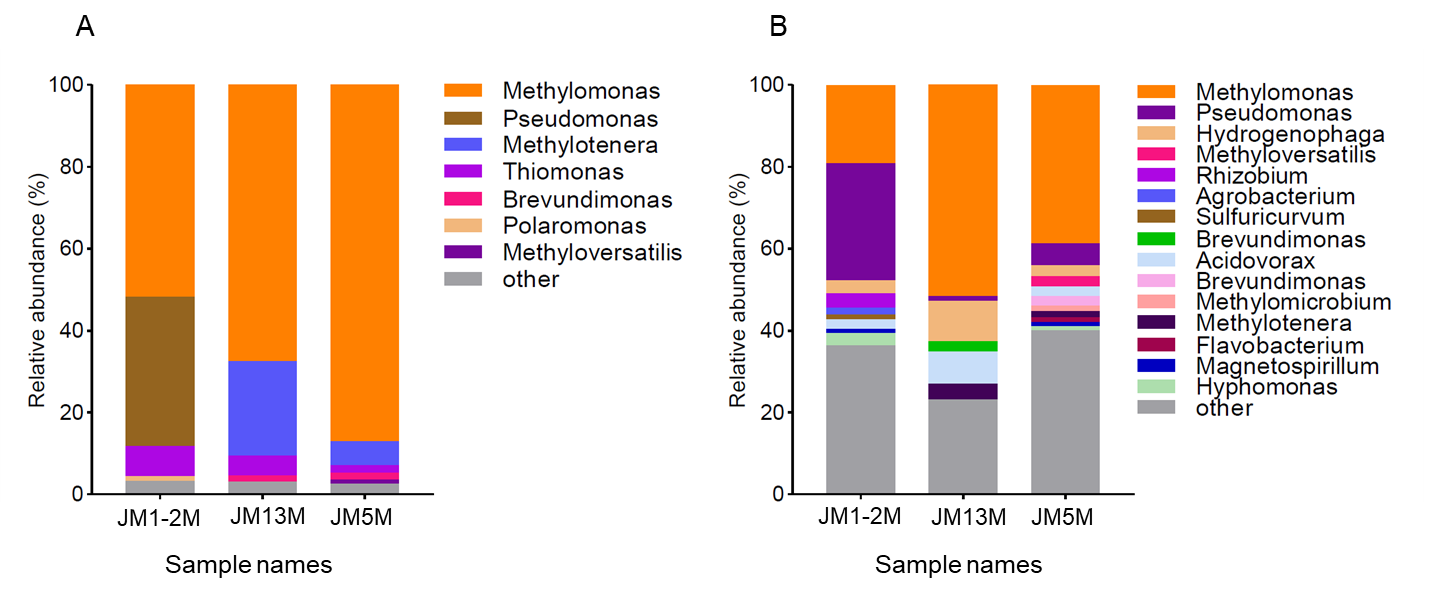


Figure S3. Taxonomic classification of metagenomic sequencing data performed using (A) MetaPhlAn2 and (B) Kraken2.


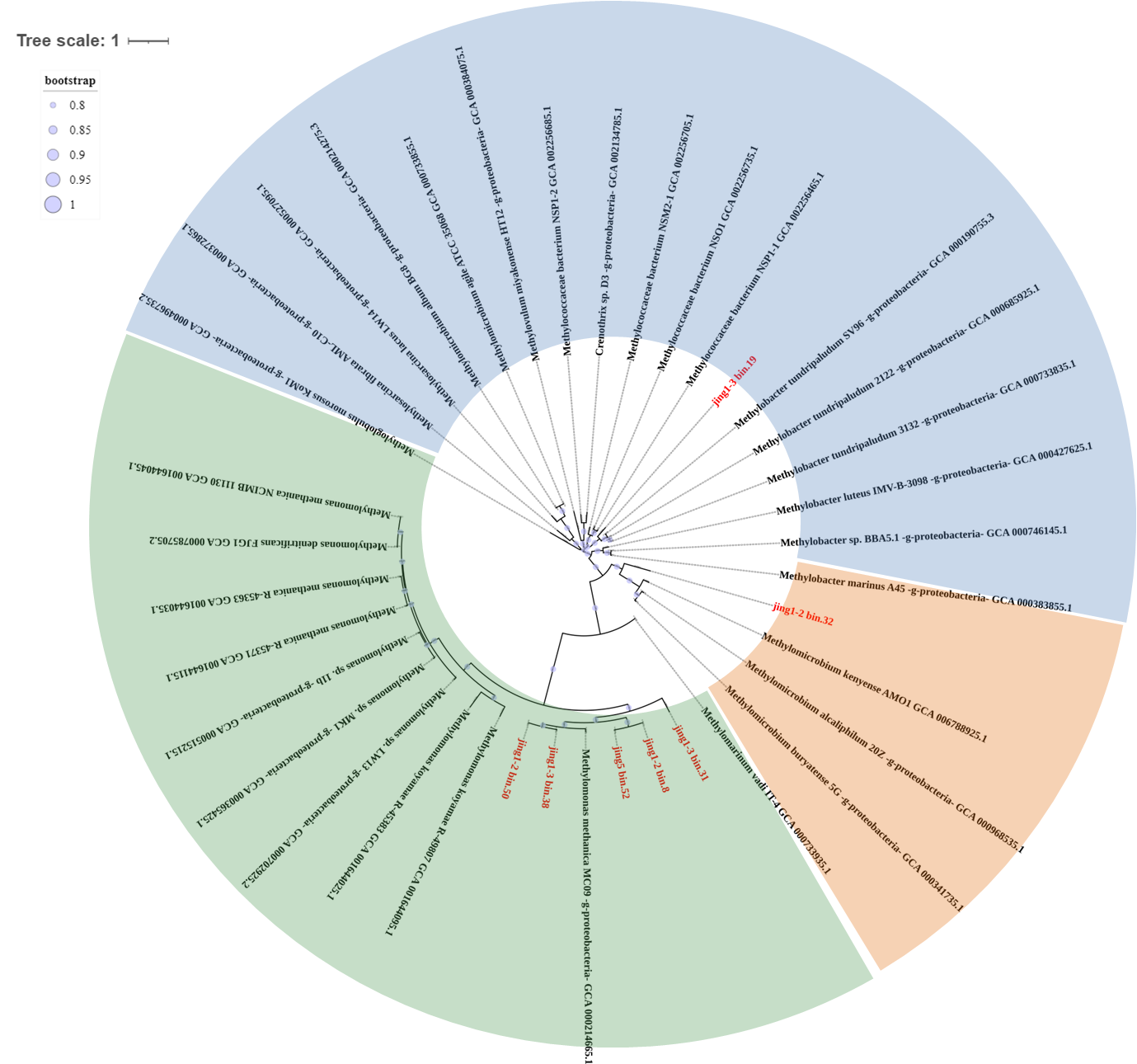


Figure S4. Phylogenetic placement of methane-oxidizing bacteria MAGs. The Maximum Likelihood tree was constructed using PhyloPhlAn pipeline which was the most accurate resulting tree of life is built using 44,600 aligned amino-acid positions sampled from 400 proteins. Bootstrap values > 0.80 are marked with grey dots.
